# [Retracted] MicroRNA‑708 inhibits the proliferation and invasion of osteosarcoma cells by directly targeting ZEB1

**DOI:** 10.3892/mmr.2023.12936

**Published:** 2023-01-09

**Authors:** Jun He, Deng Xiang, Yanshui Lin

Mol Med Rep 16: 3948–3954, 2019; DOI: 10.3892/mmr.2019.10013

Following the publication of this paper, it was drawn to the Editors’ attention by a concerned reader that cell migration assay data shown in Figs. 2C and 5D were strikingly similar to data that had appeared in different form in other articles by different authors. Owing to the fact that the contentious data in the above article had already been published elsewhere prior to its submission to *Molecular Medicine Reports*, the Editor has decided that this paper should be retracted from the Journal. The authors were asked for an explanation to account for these concerns, but the Editorial Office did not receive a reply. The Editor apologizes to the readership for any inconvenience caused.

